# Supplementary material for: Empirical methods for controlling false positives and estimating confidence in ChIP-Seq peaks
Source: BMC Bioinformatics. 2008 Dec 5;9:523. doi: 10.1186/1471-2105-9-523 (PMC2628906; doi:10.1186/1471-2105-9-523)
Supplement: Additional File 1 — A variety of html documents from the USeq web site detailing the available applications, their best usage, output file type descriptions, command line menus, etc. [file 1471-2105-9-523-S1.zip › USeqUserGuides/JavaDoc.zip!/JavaDoc/trans/roc/class-use/RocWindow.html]

Uses of Class trans.roc.RocWindow


|  |  |  |
| --- | --- | --- |
| **Overview** \| **Package** \| **Class** \| **Use** \| **Tree** \| **Deprecated** \| **Index** \| **Help** | |  |
| PREV   NEXT | **FRAMES**    **NO FRAMES**     **All Classes** |  |


---


## **Uses of Class trans.roc.RocWindow**

| Packages that use RocWindow | |
| --- | --- |
| **trans.roc** | For manipulating the BAC dmel spike in data and generating ROC curves. |

Uses of RocWindow in trans.roc

| Methods in trans.roc that return RocWindow | |
| --- | --- |
| `static RocWindow[]` | `ExtractNonOverlappingWindows.extractNonOverlappingWindows(RocWindow[] r)`             Assumes the windows are sorted |
| `static RocWindow[]` | `ParsePatternedWindows.fetchProximalWindows(GeneGroup[] geneGrps, RocWindow[] windows, int sizeUpstream)` |
| `static RocWindow[]` | `ParsePatternedWindows.filterRocWindows(RocWindow[] windows, double cutOff, boolean greaterThan)`             Scans an array of RocWindow for those that are greater than (or less than if boolean is false) or equal to the cutOff. |
| `static RocWindow[]` | `ParsePatternedWindows.loadRocWindows(java.io.File[] arrayLists)`             Loads all the RocWindows into one from serialized ArrayLists of RocWindows. |
| `static RocWindow[]` | `ParsePatternedWindows.loadTxtRocFile(java.io.File file)`             reads a txt roc window (chrom, start, stop, middle(not used), score). |

| Methods in trans.roc with parameters of type RocWindow | |
| --- | --- |
| `static RocWindow[]` | `ExtractNonOverlappingWindows.extractNonOverlappingWindows(RocWindow[] r)`             Assumes the windows are sorted |
| `static RocWindow[]` | `ParsePatternedWindows.fetchProximalWindows(GeneGroup[] geneGrps, RocWindow[] windows, int sizeUpstream)` |
| `static RocWindow[]` | `ParsePatternedWindows.filterRocWindows(RocWindow[] windows, double cutOff, boolean greaterThan)`             Scans an array of RocWindow for those that are greater than (or less than if boolean is false) or equal to the cutOff. |
| `boolean` | `RocWindow.intersects(RocWindow other)` |
| `static void` | `ParsePatternedWindows.printChroms(RocWindow[] r)` |
| `static void` | `RocWindow.printSGRWindows(RocWindow[] w)` |
| `static void` | `ParsePatternedWindows.writeSGRFile(RocWindow[] r, java.io.File file)`             Writes an sgr file. |
| `static void` | `ParsePatternedWindows.writeTxtRocFile(RocWindow[] r, java.io.File file)`             Writes a chrom, start, stop, score file. |

---


|  |  |  |
| --- | --- | --- |
| **Overview** \| **Package** \| **Class** \| **Use** \| **Tree** \| **Deprecated** \| **Index** \| **Help** | |  |
| PREV   NEXT | **FRAMES**    **NO FRAMES**     **All Classes** |  |


---
